# Effect of Ti Doping on the Microstructure and Properties of SiC_p_/Al Composites by Pressureless Infiltration

**DOI:** 10.3390/ma17071608

**Published:** 2024-04-01

**Authors:** Ruijie Feng, Haibo Wu, Huan Liu, Yitian Yang, Bingbing Pei, Jianshen Han, Zehua Liu, Xishi Wu, Zhengren Huang

**Affiliations:** 1 School of Material Science and Chemical Engineering, Ningbo University, Ningbo 315211, China; fengruijie@nimte.ac.cn; 2 Ningbo Institute of Materials Technology and Engineering, Chinese Academy of Sciences, Ningbo 315201, China; liuhuan@nimte.ac.cn (H.L.); yangyitian@nimte.ac.cn (Y.Y.); peibingbing@nimte.ac.cn (B.P.); hanjianshen@nimte.ac.cn (J.H.); liuzehua@nimte.ac.cn (Z.L.); wuxishi@nimte.ac.cn (X.W.)

**Keywords:** SiC_p_/Al, first-principles, adhesion work, Ti doping, interface wetting

## Abstract

The effects of Ti doping on the microstructure and properties of SiC_p_/Al composites fabricated by pressureless infiltration were comprehensively investigated using first-principles calculations and experimental analyses. First-principles calculations revealed that the interface wetting and bonding strength in an Al/SiC system could be significantly enhanced by Ti doping. Subsequently, the Ti element was incorporated into SiC preforms in the form of TiO_2_ and TiC to verify the influence of Ti doping on the pressureless infiltration performance of SiC_p_/Al composites. The experimental results demonstrated that the pressureless infiltration of molten Al into SiC preforms was promoted by adding TiC or TiO_2_ due to the improved wettability. However, incorporating TiO_2_ leads to the growth of AlN whiskers under a N_2_ atmosphere, thereby hindering the complete densification of the composites. On the other hand, TiC doping can improve wettability and interface strength without deleterious reactions. As a consequence, the TiC-doped SiC_p_/Al composites exhibited excellent properties, including a high relative density of 99.4%, a bending strength of 287 ± 18 MPa, and a thermal conductivity of 142 W·m^−1^·K^−1^.

## 1. Introduction

In the rapidly evolving microelectronics industry, electronic packaging materials play a crucial role. They not only support semiconductor integrated circuits (ICs) and their interconnecting circuits but also provide a reliable and stable operating environment for chips. With the development of integrated circuits towards higher integration levels and the advancement of high-density electronic packaging technology, the performance requirements for electronic packaging materials have become increasingly stringent. To meet the challenges posed by high integration and high-density packaging technologies, future electronic packaging materials must possess the following comprehensive properties [1,2,3,4]: (1) High thermal conductivity (TC). Due to the significant amount of heat generated during the operation of high-integration chips and high-density electronic packaging, high thermal conductivity is a primary requirement for electronic packaging materials. If the heat cannot be rapidly dissipated through the packaging material, it will lead to a sharp increase in the operating temperature of electronic components and chips, thereby affecting their reliability and lifespan. Research has shown that chips operating above a baseline temperature (100 °C) exhibit an exponential relationship between failure rate and operating temperature, with the failure rate increasing by 2 to 3 times for every 18 °C rise in temperature; (2) Low thermal expansion coefficient (CTE). Thermal stress caused by temperature changes is another significant challenge in the field of electronic packaging. Differences in CTE between electronic packaging materials and chip materials can lead to creep, fatigue, and even structural warping and fractures. The CTE of packaging materials needs to be matched with semiconductor materials such as silicon or gallium arsenide to minimize thermal stress. Studies have indicated that material failure can occur after 100 thermal cycles if the CTE difference between the packaging substrate and the chip exceeds 1.2 × 10^−5^ K^−1^; (3) Lightweight. Electronic packaging materials should also have a low density to suit the development trend of microelectronic devices towards high performance, being lightweight, and miniaturization. In the aerospace and portable electronic devices fields, the demand for lightweight materials is particularly urgent; (4) High strength and stiffness. Electronic packaging materials are required to support and protect chips, and hence must possess adequate strength and hardness. This ensures that the packaging materials can effectively protect chips from damage under various physical conditions; (5) Ease of mechanical processing. Materials should have a good electroplating and welding performance, conducive to processing into various complex shapes and meeting the technical requirements of the device packaging process; (6) Low cost. Only by improving the economic efficiency of materials can they have good market competitiveness, which is conducive to large-scale mass production.

Silicon carbide particle-reinforced aluminum (SiC_p_/Al) composites exhibit a range of superior properties, including a low coefficient of thermal expansion, high thermal conductivity, exceptional specific strength, and high specific modulus. These attributes make them ideally suited to meet the demanding performance requirements of advanced electronic packaging materials [5,6]. Several processes have been developed for the fabrication of SiC_p_/Al composites, mainly including pressure [7] or pressureless melt infiltration [8], extrusion casting [9], and powder metallurgy [10,11]. Among these, pressureless infiltration is an economical and efficient near-net-shape forming technique that combines material processing and part formation. However, the poor wettability between molten Al and SiC results in slow infiltration rates during pressureless infiltration, leading to prolonged interface reaction times. This inevitably leads to an increased content of brittle phases of Al_4_C_3_ [12,13], significantly affecting the overall performance of SiC_p_/Al composites [14].

To overcome these obstacles, considerable efforts have been made, such as optimizing the infiltration temperature, time, and atmosphere [5,8], pre-oxidizing SiC preforms [15,16,17,18,19], and adding active elements [15,20,21,22]. Previous studies have shown that the addition of Mg and Si elements can improve the interface structure between SiC and the Al matrix. Mg has been proven to reduce the viscosity and surface tension of aluminum, leading to the wetting of SiC by molten Al, while the addition of Si can prevent or delay the occurrence of undesirable interface reactions [23]. However, excessive addition of Mg and Si can lead to a decrease in the thermodynamic performance of the composite materials, due to the low vapor pressure of Mg and segregation of Si [24]. Beyond Mg and Si, Ti has also been demonstrated to significantly improve the wettability between ceramics and liquid metals. For example, with the addition of a small amount of Ti, the contact angle of Ag or Cu-Ag alloy on Al_2_O_3_ was reduced from well over 90° to between 10° and 20° [25]. Furthermore, Xu et al. [26] investigated the wetting and interface behavior of molten Al-Ti alloy on 4H-SiC substrates, showing that the addition of Ti can enhance the wetting between Al and SiC, leading to the elimination of the detrimental Al_4_C_3_ phase.

The aforementioned studies indicate that Ti holds promise as an active element for improving the interface structure and thermodynamic performance of SiC_p_/Al composite materials. However, much of this research has focused on the microscale impact of Ti on the wettability at the SiC interface through sessile drop methods [27]. For composite materials, the actual impact is more macroscopic, and the actual fabrication process may encounter issues such as distribution. Moreover, with the widespread application of SiC_p_/Al composite materials in the electronic packaging field, the actual performance of the materials cannot be ignored. This work aims to fill the gap in this area of research and provide new pathways for the fabrication of large volumes of high-performance SiC_p_/Al composite materials.

In this work, Ti elements were introduced into SiC in the form of TiC and TiO_2_, and SiC_p_/Al composite materials were fabricated through Ti-activated pressureless infiltration. By combining first-principles calculations with experimental analysis, the effects of Ti doping on the wettability, interface structure, microstructure, and properties of SiC_p_/Al composite materials were systematically investigated.

## 2. Materials and Methods

### 2.1. Computational Model Building

**Figure 1:**
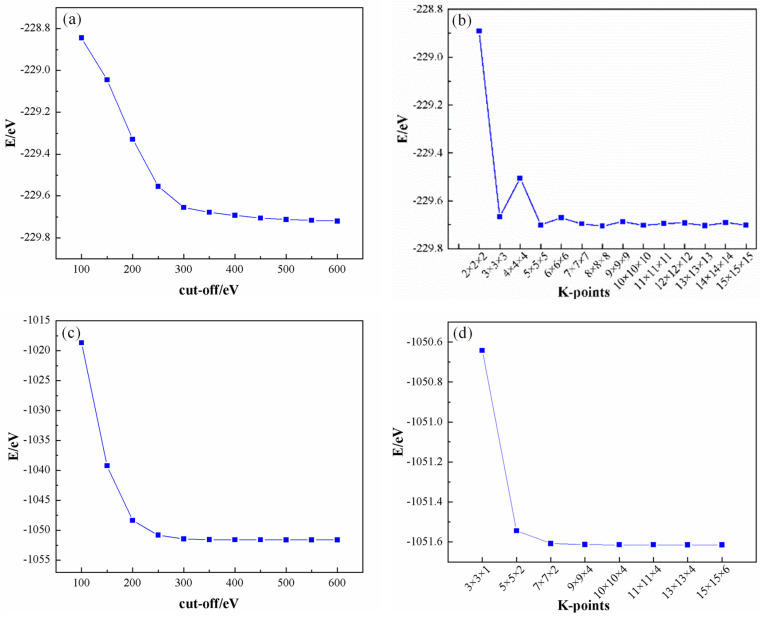
Convergences of Al and SiC: (**a**) Al cut−off; (**b**) Al K−points; (**c**) SiC cut−off, and (**d**) SiC K−points.

For the computational analyses, the Cambridge Serial Total Energy Package (CASTEP) [28] software, integrated within the Materials Studio 2020 suite, was utilized to perform the calculations. Employing the Perdew–Burke–Ernzerhof (PBE) exchange-correlation functional under the generalized gradient approximation (GGA) framework facilitated meticulous consideration of the correlation effects. As illustrated in Figure 1, a convergence criterion was established whereby the plane wave truncation energy (cut-off) was set at 400 eV, ensuring that the energy convergence threshold was achieved at 1 × 10^−5^ eV/atom, which corresponds to 9.6485 × 10^4^ J/mol. The sampling of the Brillouin zone was conducted using the Monkhorst–Pack k-point grid method, with Al utilizing a 13 × 13 × 13 grid and SiC employing a 10 × 10 × 4 grid, respectively. All computational parameters were adjusted to ultra-fine settings to ensure the precision of the results. The ultrasoft pseudopotential approach was employed to accurately describe the interactions between ions and valence electrons. Prior to the construction of the interface model, each cell structure was subjected to optimization based on the optimized structures to ensure the stability and accuracy of the resultant interface model.

The molecular structure models for Al and 4H-SiC were sourced directly from the Materials Studio 2020 software library. After their importation, these Al and 4H-SiC cells were subjected to optimization leveraging the aforementioned computational methodologies and parameters. The outcomes of these optimizations are succinctly documented in Table 1 for aluminum and Table 2 for 4H-SiC, detailing the optimized structural parameters and relevant physical properties derived from the computational analysis. 

In the construction of the surface structure model, Al and 4H-SiC are oriented along their (111) and (0001) crystal planes, respectively. These orientations are selected due to their representation of the close-packed planes within Al and 4H-SiC, a characteristic that confers the lowest energy among all low-index crystal planes. This structural alignment facilitates the formation of an energetically favorable Al(111)/SiC(0001) two-phase interface, offering an optimal configuration for examining interfacial properties. 

To ensure the structural model accurately represents the physical characteristics of the interface, a convergence test concerning the thickness of the surface layers is imperative. This test is particularly crucial for the Al(111) surface model, where the objective is to identify a thickness that reflects the stable, bulk properties of the material with minimal computational resources. The convergence of surface energy, a critical parameter in assessing the stability of the surface model, is calculated using the formula

(1) $$E_{surf}=\frac{E_{slab}-(\frac{N_{slab}}{N_{bulk}})E_{bulk}}{2A}$$

where *E_surf_* represents the total energy of the system incorporating a vacuum layer introduced after the section, *N* denotes the number of atoms, *E_bulk_* signifies the power of the primitive cell, and *A* corresponds to the surface area of the model.

**Figure 2:**
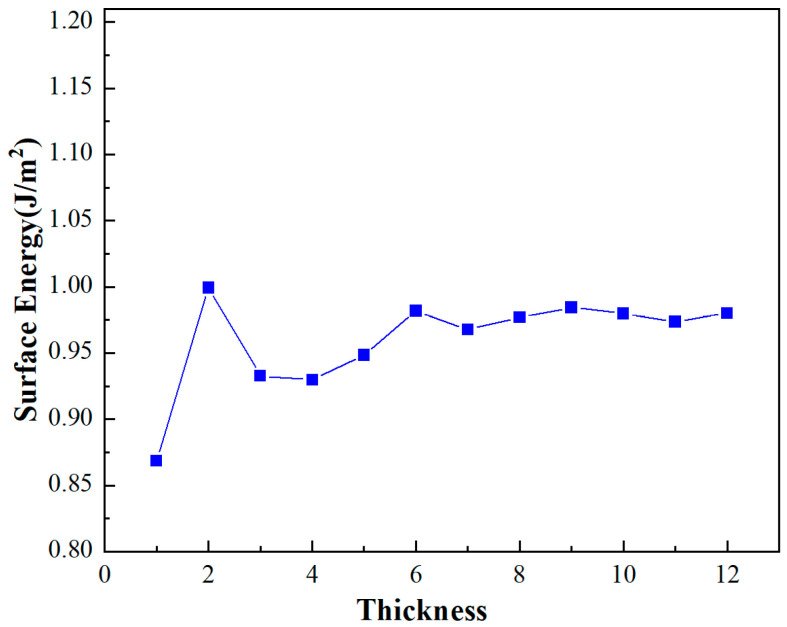
Surface energy of Al(111).

The analysis of the Al(111) surface model reveals that variations in surface energy stabilize beyond a thickness of 7 atomic layers in Figure 2. This stabilization indicates that the system’s energy properties converge at this thickness, thereby making it an appropriate choice for subsequent computational evaluations. Consequently, a structure comprising a 7-layer thickness is adopted for the detailed investigations to follow. This thickness ensures a balance between computational efficiency and the accuracy of the model’s representation of real-world physical properties. 

**Figure 3:**
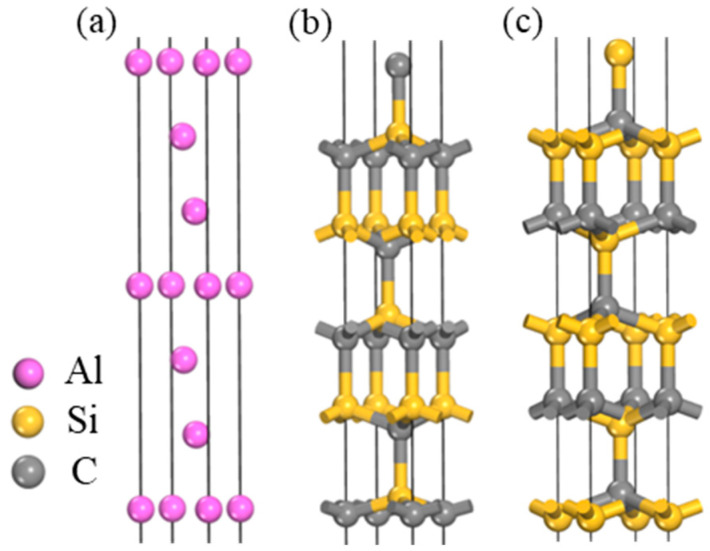
Surface structure model: (**a**) Al; (**b**) C is used as the termination surface of SiC; (**c**) Si is used as the termination surface of SiC.

The determination of the surface thickness of SiC(0001) in this study was based on the convergence of atomic relaxation on the SiC surface. Convergence was achieved when increasing the thickness of the SiC(0001) surface model resulted in an equal amount of relaxation in the atomic layer spacing compared to the previous surface and negligible relaxation between intermediate nuclear layers. Table 3 presents the ratio of 4H-SiC layer spacing for different surface thicknesses. When the thickness ≥11, there was a tendency for convergence in variations in SiC(0001) surface atomic layer spacing, with almost zero atom relaxation observed in the central layer. Therefore, an 11-layer thickness of SiC(0001) was selected. The surface models of Al(111) and SiC(0001) crystal faces are depicted in Figure 3. On the SiC(0001) surface, there exist two surface models with either Si or C as the terminating species. However, based on the research conducted by Wang [33] and Zhang [34] et al., as well as considering the tendency of the interface reaction 4Al + 3SiC = Al_4_C_3_ + 3Si to occur at the grain boundary, it can be inferred that Al atoms exhibit a preference for bonding with C atoms. Consequently, the interface formed by the surface model with C as the terminating species exhibits a higher interfacial bonding strength. Therefore, to establish the interface model, we selected Al(111) and SiC(0001) surfaces terminated by C.

**Figure 4:**
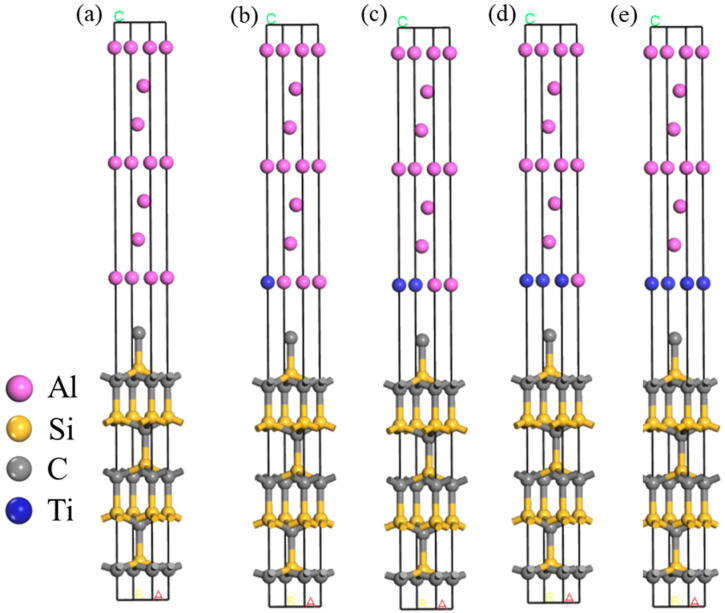
Schematic diagram of Ti doping: (**a**) undoped, (**b**) 1 Ti, (**c**) 2 Ti, (**d**) 3 Ti, (**e**) 4 Ti.

The established Al(111)/SiC(001) interface model is depicted in Figure 4a. Upon calculation, the degree of mismatch for this interface model is determined to be 1.15%. According to Hong et al. [35], when there is a minimal mismatch at the interface, the resulting strain becomes negligible, thus indicating a fully matched condition.

### 2.2. Comparison Experiments

The experiment used 50 μm, 5 μm SiC, 1 μm TiC, and TiO_2_, four raw materials with more than 99% purity, to prepare the preform. SiC, TiC, TiO_2_, organic additives, and pore-forming agents were mixed proportionally by high-speed ball milling (TiC and TiO_2_ addition amounts were 5 wt%). The mixture was then dried and pressed. TiO_2_ and TiC were added to the SiC preform because of the high melting point of Ti, which cannot be melted when added to the aluminum alloy. In addition, both TiC and SiC are carbides, which have a good chemical stability when added to SiC preforms. To evaluate the improvement of the wettability of the composites by introducing Ti as a penetration promoter, the same composition and process parameters were used to prepare the control samples without Ti to eliminate other influencing factors. The samples without Ti were labeled as the C group, the samples with TiO_2_ were labeled as the TO group, and the samples with TiC were labeled as the TC group. The three groups of samples were all sintered at 2423 K in an argon atmosphere to prepare porous preforms and then oxidized at 1473 K. Al-7%Mg (by weight) aluminum alloy was synthesized in laboratory conditions with pure Al and pure Mg with a purity ≥99%. 

The pressureless infiltration was performed in a high-temperature horizontal vacuum sintering furnace. The oxidized SiC porous preform was placed at the bottom of the graphite crucible, and the aluminum alloy was placed at the top of the SiC porous preform. Then, the pressureless infiltration was performed at 1223 K for 4 h in a N_2_ atmosphere.

### 2.3. Test and Characterization

The density (D) of the sample was determined using the Archimedes method. The relative density (RD) represents the ratio of the density to the theoretical density, while the theoretical density was calculated based on the mixture rule. The phase composition of the sample was analyzed using an X-ray diffractometer (ADVANCED8, Burkle, Freudenstadtcity, Germany). Additionally, field emission scanning electron microscopy (SEM, S-8230, Hitachi, Marunouchi, Japan) was employed to observe the fracture morphology of sintered samples. The thermal conductivity of the sample was measured utilizing a laser thermal conductivity meter (LFA467, NETTSCH, Selb, Germany), with samples sized 10 mm × 10 mm × 3 mm. 

## 3. Results and Discussions

### 3.1. Effect of Ti Doping on the Adhesion Work of the SiC_p_/Al Interface

**Figure 5:**
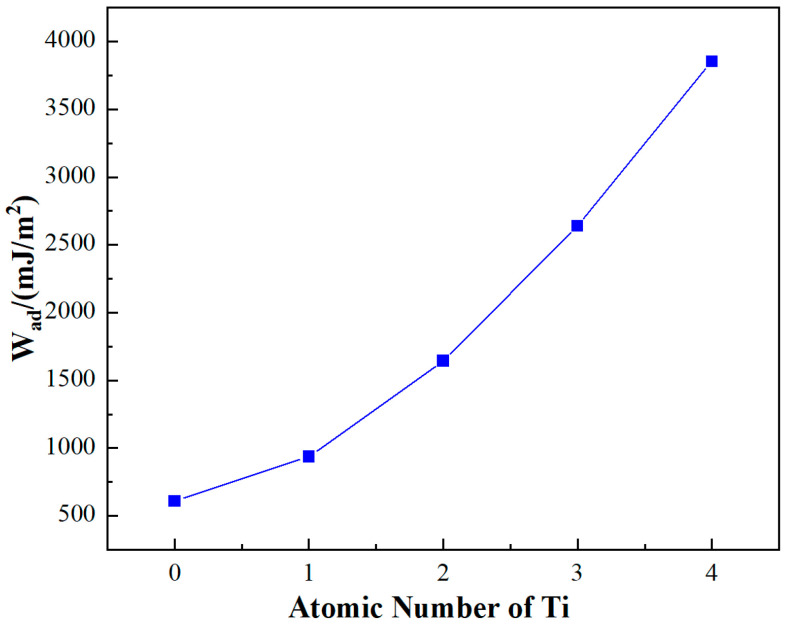
Influence of Ti doping on the adhesion work of the SiC_p_/Al system.

The exploration into the effects of Ti doping on the interface of the SiC/Al system yielded significant insights, particularly concerning the adhesion work (W_ad_) which serves as a measure of the interface energy. As depicted in Figure 5, substituting four surface Al atoms with Ti atoms markedly influences the adhesion work. Initially, the undoped SiC_p_/Al system demonstrates a W_ad_ value of 609.80 mJ/m^2^. With the progressive incorporation of Ti atoms, a near-linear augmentation in W_ad_ is observed. Remarkably, upon the introduction of four Ti atoms, W_ad_ escalates to 3852.95 J/m^2^, registering an enhancement of approximately 432%.

This substantial increase in adhesion work due to Ti doping can be interpreted through the lens of energetic interactions at the interface. W_ad_ is fundamentally the energetic cost per unit area to detach two phases at their interface, representing the change in free energy before and after adhesion. This concept is pivotal across various interfacial phenomena, encompassing interactions between liquid–liquid, liquid–solid, and gas–solid phases. Specifically, in the context of the SiC_p_/Al system, wetting—a subset of adhesion—pertains to the interface between a liquid and a solid phase, with the soaking work offering a quantitative measure of the adhesive force at the interface.

Thus, the observed increase in W_ad_ upon Ti doping not only underscores an enhanced adhesion (or bonding strength) at the SiC_p_/Al interface but also signifies improved wettability. These findings suggest that Ti doping facilitates a stronger and more energetically favorable interaction between the SiC particles and the Al matrix.

### 3.2. Microstructure and Phase Composition of SiC Preforms

**Figure 6:**
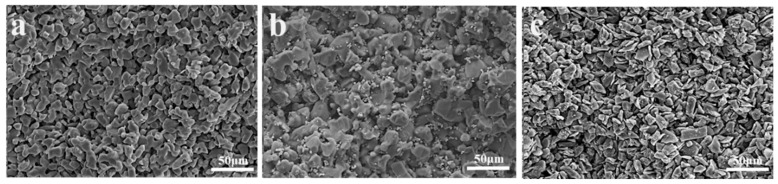
SEM images of different SiC preforms: (**a**) C; (**b**) TO; and (**c**) TC.

**Figure 7:**
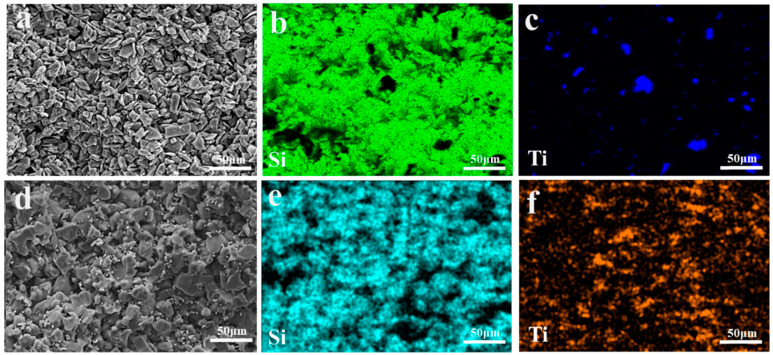
EDS mappings of different SiC preforms: (**a**–**c**) TO and (**d**–**f**) TC.

The porosity test results of three groups of preforms, namely C, TO, and TC, are presented in Table 4. The observed 7% difference in porosity between the C and TC groups can be attributed to the reduction in porosity caused by the abnormal grain growth of TiC during the sintering process of SiC preforms. The microstructure of the preform is depicted in Figure 6. No significant disparity is observed in other aspects between the preforms with and without Ti addition. In all three groups, SiC particles exhibit uniform distribution, coarse grains form the framework, and refined grains disperse within the gaps among larger grains, thereby establishing the skeletal structure of the SiC preform. The elemental distribution of the preform is illustrated in Figure 7. The TO sample containing TiO_2_ demonstrates well-dispersed elements without any noticeable segregation phenomenon; conversely, slight agglomeration of titanium elements at a micro level can be observed in the TC preform having TiC while maintaining macro-level uniformity.

**Figure 8:**
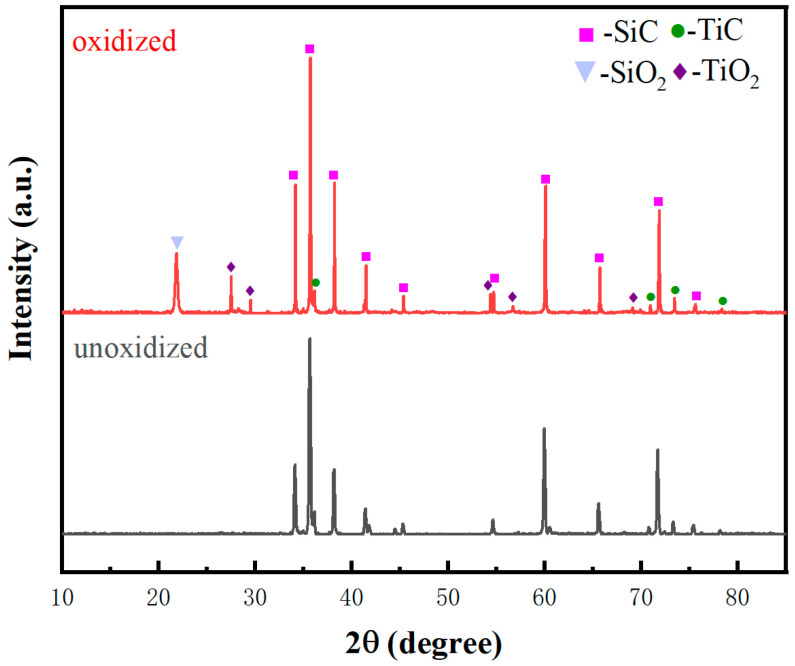
XRD patterns of the TC preform before and after oxidation.

The oxidation treatment of the preform primarily aims at surface modification, resulting in the formation of partial SiO_2_, which significantly enhances the material’s wettability [15,16,17,18,19,32,36]. Oxidation treatment of TC can also be employed to improve wettability by inducing partial TiO_2_ formation. Figure 8 presents the XRD analysis of the TC before and after oxidation, demonstrating the generation of SiO_2_ and TiO_2_. TiC is also residual, confirming the achievement of desired effects through oxidation treatment.

### 3.3. Effect of Ti Doping on Properties of SiC_p_/Al Composites

The composite performance presented in Table 5 demonstrates a significant enhancement in the relative density of the composites following the incorporation of TiO_2_ and TiC, thereby substantiating their crucial role in improving material wettability. However, when compared to TiO_2_, the sample containing TiC exhibits a density close to the theoretical density of the material, indicating a more pronounced effect shown by TiC. As neither C nor TO samples exhibit densification, mechanical and thermal property testing was solely conducted on the TC sample. The test results revealed excellent thermal and mechanical properties for TC.

**Figure 9:**
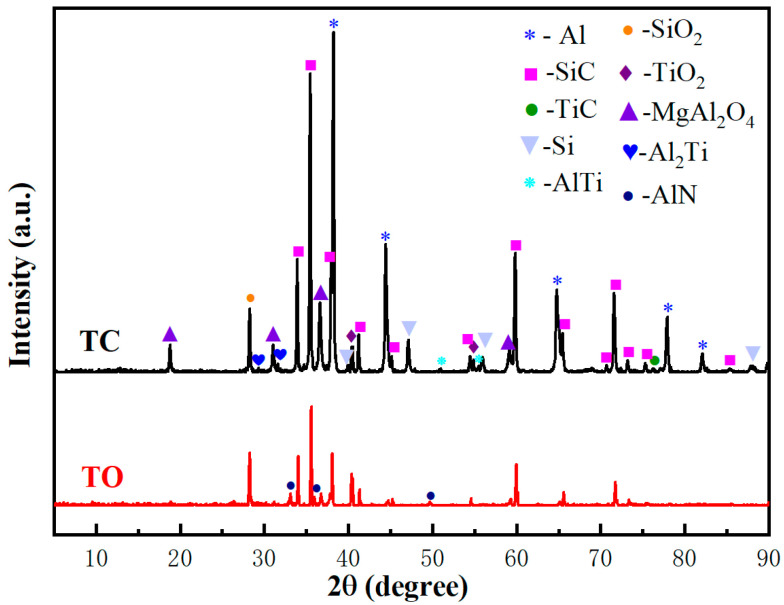
XRD patterns of the TO and TC composites.

The XRD patterns of the TC and TO composites in Figure 9 reveals the absence of Al_4_C_3_, with SiC and Al being the predominant phases. Eight other phases are also present, including TiC, TiO_2_, SiO_2_, Si, AlTi, Al_2_Ti, AlN, and MgAl_2_O_4_. Through comprehensive analysis of all potential reactions taking place at the interface, it can be deduced that the predominant chemical reactions are as follows:

TiC(s) + 2O_2_ = TiO_2_(s) + CO_2_ (2)

SiC(s) + 2O_2_ = SiO_2_(s) + CO_2_ (3)

3Mg(l) + 4Al_2_O_3_(s) = 3MgAl_2_O_4_(s) + 2Al(l) (4)

Mg(l) + 2Al(l) + 2O_2_ = MgAl_2_O_4_(s) (5)

Mg(l) + 2Al(l) + 2SiO_2_(s) = MgAl_2_O_4_(s) + 2Si(s) (6)

3TiO_2_(s) + 7Al = 2Al_2_O_3_(s) + 3AlTi(s) (7)

3TiO_2_(s) + 10Al = 2Al_2_O_3_(s) + 3Al_2_Ti(s) (8)

2Al(l) + N_2_(g) = 2AlN(s) (9)

Reactions (2) and (3) predominantly occurred during the oxidation pretreatment stage of the preform, while Responses (4)–(9) primarily took place during the infiltration stage, with Reaction (9) was exclusively observed in the TO sample.

**Figure 10:**
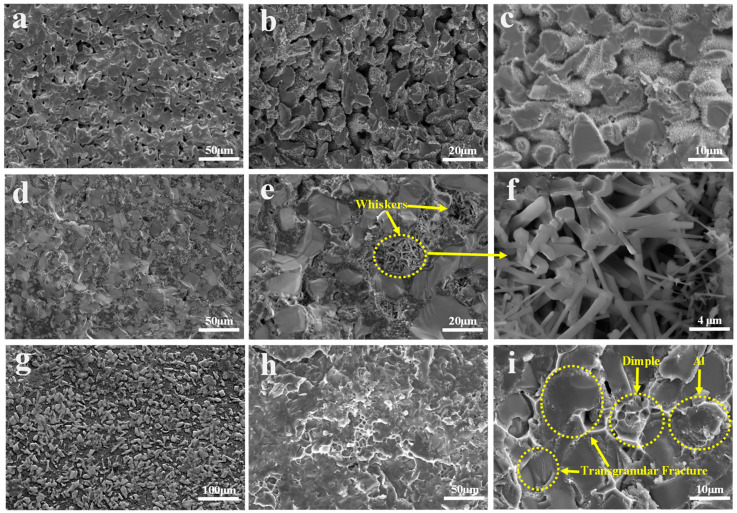
SEM images of the SiCp/Al composites: (**a**–**c**) SEM of C; (**d**–**f**) SEM of TO; (**g**–**i**) SEM of TC.

**Figure 11:**
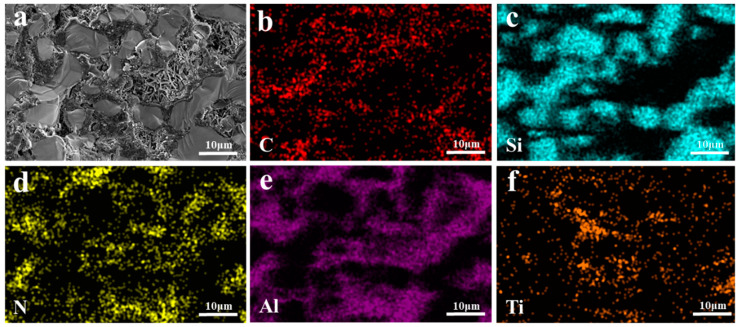
EDS mappings of TO composite materials: (**a**) SEM image; (**b**)C; (**c**) Si; (**d**) N; (**e**) Al; (**f**) Ti.

The SEM patterns of the composite material are presented in Figure 10. By comparing the SEM images of C, TO, and TC composites, it is evident that the porosity of the C sample is higher, and molten Al does not fully infiltrate the SiC skeleton; at the same time, a small amount of Al particles are adsorbed on the surface of the SiC particles. However, these samples fail to achieve complete compactness due to inadequate wettability or the short immersion time during the preparation process. The pores in the TO sample are noticeably reduced but accompanied by whisker formation within them. Figure 11 reveals that these whiskers exist in both C and TC samples. This observation suggests that a high TiO_2_ content may facilitate AlN whisker growth under N_2_ atmosphere conditions. These grown AlN whiskers impede Al infiltration pathways due to their existence in the solid state, resulting in increased material porosity. Conversely, the TC sample with TiC addition exhibits complete compaction without noticeable pores, presenting an interleaved and evenly distributed structure of SiC and Al phases. Al fully encapsulates SiC particles. At this stage, the interfacial bonding strength between SiC particles and Al surpasses the intrinsic strength of SiC particles, resulting in a predominantly transgranular fracture mode. Ductile dimple fractures are observed during fracture, indicating robust bonding between adjacent Al particles. SEM images demonstrate that TiC addition enhances wettability and interface bonding strength between SiC_p_/Al phases, thereby improving mechanical properties.

**Figure 12:**
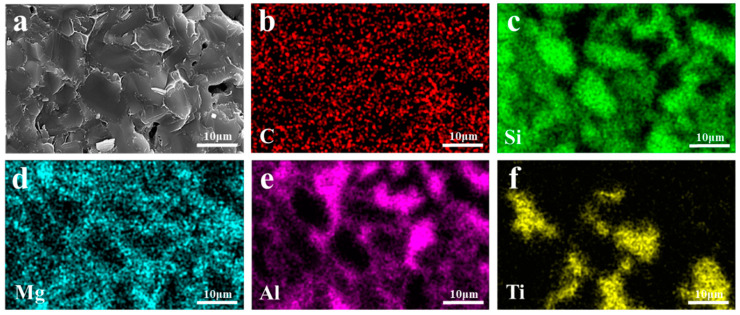
EDS mappings of TC composite materials: (**a**) SEM image; (**b**) C; (**c**) Si; (**d**) Mg; (**e**) Al; (**f**) Ti.

EDS images of the TO and TC composites are presented in Figure 11 and Figure 12, respectively. All elements in TO and TC exhibit uniform distribution without evident agglomeration or segregation. Specifically, SiC and Al intertwine to form a cross-penetration structure. In the TO sample, the whiskers existing in the holes are AlN whiskers based on the N element distribution. These solid whiskers impede the infiltration process, leading to a reduction in material density. On the other hand, in the TC sample, the TiC component remains unchanged and is distributed within the SiC framework; however, some of it transforms TiO_2,_ compounds of which subsequently participate in Reactions (6) and (7), resulting in the formation of AlTi and Al_2_Ti phases that coexist with the aluminum matrix. Notably, incorporating TiC raw materials into the SiC framework compositionally hinders the generation of the Al_3_Ti phase at interfaces. Instead, interface products such as AlTi and Al_2_Ti are formed due to restricted mutual diffusion between the liquid-phase alloy and Ti caused by the presence of SiC particles locally reducing the aluminum concentration below levels required for creating the TiAl_3_ phase, as depicted in Figure 12e,f.

The results demonstrate that Ti doping significantly influences the properties of pressureless infiltration SiC_p_/Al composites. This contribution effectively enhances the wettability and interface bonding strength of the system while also positively impacting its mechanical and thermal properties. The improvement in material properties due to Ti doping can be attributed to five main factors [36,37]: (1) Chemical reactivity. Both TiO_2_ and TiC can chemically react with aluminum to form new phases at the interface. These reactions can modify the surface energy and reduce the interfacial tension, thereby improving the wetting of the molten aluminum on the SiC particles. (2) Formation of active interfaces. The introduction of TiC and TiO_2_ leads to the formation of active interfaces that are more conducive to wetting. TiC, in particular, can act as a catalyst for wetting, reducing the contact angle between the molten aluminum and the SiC. The Ti atoms play a crucial role in this process by altering the electronic properties at the interface, enhancing the interaction between the molten metal and the ceramic phase. (3) Thermodynamic stability. The presence of TiO_2_ and TiC can lead to the formation of thermodynamically stable phases at the interface, which can further improve wettability. These stable phases can prevent the formation of undesirable compounds that could hinder wetting and adhesion. (4) Microstructural effects. The addition of TiO_2_ and TiC can influence the microstructure of the interface, creating conditions that are more favorable for wetting. For example, the formation of fine Ti-containing phases can provide additional pathways for the flow of molten aluminum, facilitating the infiltration process. (5) Modification of surface and interfacial energies. The introduction of TiO_2_ and TiC modifies the surface and interfacial energies of the SiC particles and the aluminum matrix, respectively. This modification can lead to a more favorable thermodynamic condition for wetting, as characterized by a lower contact angle.

Compared to TiO_2_, incorporating TiC yields superior material properties since adding TiO_2_ leads to the growth of AlN whiskers under N_2_ atmosphere conditions, which obstructs material infiltration pathways and reduces relative density.

## 4. Conclusions

In summary, the effect of Ti doping on the properties of SiC_p_/Al materials was systematically investigated using a first-principles approach and pressureless infiltration method. The influence of different methods for introducing Ti on material performance was also examined. The research findings can be summarized as follows: 

(1) First-principles calculations revealed that Ti doping significantly enhances interface adhesion work and bonding strength, indicating its theoretical significance in facilitating the pressureless infiltration process;

(2) Experimental results indicate that the presence of Ti significantly enhances both the relative density and permeation rate of the materials. The incorporation of TiC and TiO_2_ contributes to an increased relative density in SiC_p_/Al composites, largely due to a combination of factors including chemical reactivity, the formation of active interfaces, thermodynamic stability, microstructural effects, and the modification of surface and interfacial energies. However, the formation of AlN whiskers poses challenges in achieving full densification in TiO_2_-doped samples. On the other hand, doping with TiC effectively reduces the interfacial energy between SiCp and Al and the surface tension of the Al melt, significantly improving wettability and interface strength. This enhancement in turn shortens the interface reaction time and effectively prevents the formation of Al_4_C_3_. Comparatively, TiC emerges as a superior doping agent;

(3) Characterization of the fabricated composite materials revealed that SiC_p_/Al composites doped with TiC exhibit a relative density of up to 99.4%, a bending strength of 287 ± 18 MPa, and a thermal conductivity of 142 W·m^−1^·K^−1^, underscoring TiC doping as an advantageous strategy for enhancing composite material performance.

## Figures and Tables

**Table 1:** Geometry optimization results of Al.

Source	Lattice Constant a (Å)	Lattice Constant c (Å)	Deviation (%)
References	4.05 [29]	4.05 [29]	
	4.04 [30]	4.04 [30]	
Model	4.0495	4.0495	
Calculation	4.049443	4.0494	0.0025

**Table 2:** Geometry optimization results of SiC.

Source	Lattice Constant a (Å)	Lattice Constant c (Å)	Deviation (%)
References	3.093 [31]	10.125 [31]	
	3.073 [32]	10.053 [32]	
Model	3.078003	10.046	
Calculation	3.083163	10.091233	0.7873

**Table 3:** The ratio of 4H-SiC layer spacing for different surface thicknesses.

Interlamellar Spacing (%)	Thickness			
	7	9	11	13
Δ1/2	−0.06627	0.012195	0.012725	−0.03128
Δ2/3	0.037935	−0.00529	−0.00529	0.03277
Δ3/4	−0.00901	0.007941	0.00847	−0.00424
Δ4/5	0.016913	−0.01001	−0.00948	0.005269
Δ5/6	−0.00635	0.011665	0.012195	−0.00212
Δ6/7	0.002634	−0.0074	−0.00529	0.003171
Δ7/8		0.039704	0.009529	−0.00212
Δ8/9		−0.03846	−0.01159	0.002107
Δ9/10			0.036585	−0.00212
Δ10/11			−0.03383	0.0037
Δ11/12				−0.00318
Δ12/13				0.006849

**Table 4:** Density and porosity of the SiC preform.

Samples	Density/g·cm^−3^	Porosity/%
C	1.98	39
TO	2.05	38
TC	2.25	32

**Table 5:** Properties of SiC_p_/Al composites.

Samples	Density (g·cm^−3^)	Relative Density (%)	Bending Strength (MPa)	Thermal Conductivity (W/(m·K))
C	2.6	87.2	-	-
TO	2.83	93.1	-	-
TC	3.06	99.4	287 ± 17.8	142

## Data Availability

Data are contained within the article.
